# Malaria (Poetry)

**Published:** 1881-02

**Authors:** Isabel H. Reid


### MALARIA.

(From Puck.)

Our baby lay in its mother's arms,
All sweet with its tiny dimpled charms;
But little mouth and tongue were sore,
And of its food 'twould take no more.
The Doctor hemm'd and shook his head,
And looking wise, he gravely said:
"Malaria—'tis plainly seen—
Three times a day give him quinine!"
Said Grandmamma: "Dear me, that's new;
When I was young we called it 'sprue'!"

Our urchin, Tom, ne'er off his feet,
One day his dinner could not eat;
His head ached so, he was so ill,
Poor Mother's heart with fear did fill.
The Doctor felt his hands and head,
And looking wise, he gravely said:
"Malaria—'tis plainly seen—
Three times a day give him quinine!"
Said Grandmamma: "That can't be so!
He has been smoking, sir, I know!"

Our lady Maud, at seventeen—
As bright a girl as e'er was seen—
One day turned languid, white and frail,
And roses red did strangely pale.
The Doctor felt her pulse and said,
While wisely he did shake his head:
"Malaria—'tis plainly seen—
Three times a day give her quinine!"
Said Grandmamma: "That can't be right!
Why, my good sir, she danced all night!"

Our pride, our oldest, Harry dear,
One night did act so strange and queer
That Mother, frightened, panting, said:
"Run for the Doctor—he'll be dead!"
The Doctor came, and shook his head,
And looking at him, grandly said:
"Malaria—'tis plainly seen—
Three times a day give him quinine!"
"What stuff!" said Grandma: "I am thinking
That good-for-nothing boy's been drinking!"

The head of the house, forever well,
One day fell ill, and, sad to tell,
Could not arise, but loud did cry:
"If this keeps on, I'd rather die!"
The Doctor came, stood by the bed,
And, looking solemn, gravely said:
"Malaria—'tis plainly seen—
Three times a day give him quinine!"
Growled Grandmamma: "Oh, fiddle-de-dee!
He's only bilious—seems to me!"

One day our Grandpa—eighty-four—
Complained that he could see no more;
That, at his age, it worried him
That his good eyesight should grow dim.
"I've often seen it act that way,"
The Doctor, solemnly did say.
"Malaria—'tis plainly seen—
Three times a day, sir, take quinine!"
But Grandma cried: "I never see!
Old man, you're growing old like me!"

ISABEL H. REID.

